# D-Dimer Use and Pulmonary Embolism Diagnosis in Emergency Units: Why Is There Such a Difference in Pulmonary Embolism Prevalence between the United States of America and Countries Outside USA?

**DOI:** 10.1371/journal.pone.0169268

**Published:** 2017-01-13

**Authors:** Gilles Pernod, Jeffrey Caterino, Maxime Maignan, Cindy Tissier, Jeannine Kassis, John Lazarchick, On behalf of DIET study group

**Affiliations:** 1 Department of Vascular Medicine, Grenoble-Alpes University Hospital, Université Grenoble Alpes CNRS / TIMC-IMAG UMR 5525 / Themas, Grenoble, France; 2 Department of Emergency Medicine, Ohio State University, Columbus (Ohio); United States of America; 3 Department of Emergency Medicine, Grenoble-Alpes University Hospital, Grenoble, France; 4 Department of Emergency Medicine, University Hospital Dijon, Dijon, France; 5 Department of Hematology, Hopital Maisonneuve-Rosemont, University of Montreal, Montreal, Canada; 6 Pathology and Laboratory Medicine, Medical University South Carolina, Charleston, United States of America; Kurume University School of Medicine, JAPAN

## Abstract

**Objective:**

Although diagnostic guidelines are similar, there is a huge difference in pulmonary embolism (PE) prevalence between the **United States of America** (US) and countries outside the USA (OUS) in the emergency care setting. In this study, we prospectively analyze patients’ characteristics and differences in clinical care that may influence PE prevalence in different countries.

**Methods:**

An international multicenter prospective diagnostic study was conducted in a standard-of-care setting. Consecutive outpatients presenting to the emergency unit and suspected for PE were managed using the Wells score, STA-Liatest^®^ D-Dimers and imaging.

**Results:**

The prevalence of PE in the study was 7.9% in low and moderate risk patients. Among the 1060 patients with low or moderate pre-test probability (PTP), PE prevalence was four times higher in OUS (10.7%) than in the US (2.5%) (P < 0.0001). The mean number of imaging procedures performed for one new PE diagnosis was 3.3 in OUS vs 17 in the US (P < 0.001). Stopping investigation in the case of negative D-dimers (DD combined) with low/moderate PTP was more frequent in OUS (92.7%) than in the US (75.7%) (P < 0.01). Moreover, the use of imaging was much higher in the US (44.4% vs 19.2% in OUS) in the case of moderate PTP combined with negative DD.

**Conclusion:**

Differences between US and OUS PE prevalence in emergency setting might be explained by differences in patients' characteristics and mostly in care patterns. US physicians performed computed tomographic pulmonary angiography more often than in Europe in cases of low/moderate PTP combined with negative DD.

**Trial Registration:**

ClinicalTrials.gov NCT01221805

## Introduction

Pulmonary embolism (PE) is a life-threatening condition associated with high mortality [1, 2]. Its diagnosis remains highly challenging, and the use of diagnostic tests has dramatically increased. During the period 1997–2000, the number of evaluations for PE multiplied by a factor of 6, mainly due to the widespread use of computed tomographic pulmonary angiography (CTPA), although the prevalence of PE in the emergency department decreased over the same period [3, 4], being as low as 5% in some studies from North America [5].

Several guidelines have been proposed to improve the investigation of suspected pulmonary embolism [6, 7]. Clinical prediction rules, i.e. Wells [8] and Geneva [9] scores, which are used to estimate the pretest probability (PTP) of PE, were recommended as the first step prior to ordering complementary exams. Based on these scores, it is recommended that CTPA should not be performed in patients with low/intermediate pretest probability (PTP) and a negative D-dimer (DD) [7, 10]. However, reports in US hospital and emergency departments have shown that CTPA is performed in up to 38% to 55% of patients with low PTP, even though such patients have not undergone DD testing or had a negative DD [11, 12]. In this context, the prevalence among those evaluated for PE was reported to be as low as 5% to 10% and one-third of patients were found to have undergone repeated CTPA [13]. In contrast, PE prevalence among those evaluated for the disease was found to be around 20% to 30% in Europe [14, 15]. In a post-hoc analysis of three diagnosis studies in Europe and the USA, such differences in PE prevalence were reported to be associated with specific characteristics of these two populations [16].

According to the latest requirements of the Food and Drug Administration (FDA) in the USA, and to be compliant with the new Clinical and Laboratory Standards Institute guideline regarding “Quantitative D-dimer for the Exclusion of Venous Thromboembolic Disease » [17], we conducted the DIET study, an international multicenter (Europe, Canada and USA) prospective study to validate the clinical performance of the STA^®^—Liatest^®^ D-Dimer assay when used in combination with PTP for PE exclusion [18]. In the present manuscript we report and analyze the differences in clinical care leading to the huge difference in observed PE prevalence between the different countries.

## Materials and Methods

The present report is a post hoc analysis of the DIET study. The DIET PE study was an international multicenter prospective, non-randomized, non-interventional diagnostic accuracy study (NCT01221805) in a standard-of-care setting, conducted in consecutive outpatients presenting to the emergency unit or outpatient clinic and suspected of having PE. Patients were recruited from nine different centers, located in Canada (one center), France (two centers), Italy (two centers), Spain (two centers) and the USA (two centers) (see S1 Appendix). According to the protocol, consecutive patients suspected of having PE (hemoptysis, lung-related chest pain or dyspnea), younger than 80 years, without cancer or surgery within the previous month, were managed using the Wells score, STA^®^—Liatest^®^ D-Dimers (for low and moderate pretest clinical probability only) and imaging (especially CTPA), according to local practice. According to the requirements of the FDA, the population of interest was limited to patients with low or moderate PTP. Reference PE status was determined as follows: patients with low or moderate PTP level (determined by Wells Score) were tested for D-Dimer. If the test result was positive (DD +, i.e > 500 ng/ml) the patient was sent to imaging, and his/her reference status was determined by the imaging outcome. If the test result was negative (DD-) the patient was sent to follow-up of three months. Sometimes during the follow up image was done. In this case image result was taken as reference. If during the three months of follow up there was no clinical need for imaging and no hospitalizations or other PE related symptoms, the patient reference was determined as negative.

### Ethics statement

This study was conducted in accordance with the ethical principles of the Declaration of Helsinki and in compliance with the US Code of Federal Regulation Title 21, Parts 50, 54 and 56. In addition, the study complied with elements relevant to the use of in vitro diagnostic products within the ISO 14155 “Clinical investigation of medical devices for human subjects—Good clinical practice” and ICH E6 “Good clinical practice: consolidated guideline”. The DIET Study protocol was submitted and approved by Ethics Committee or Institutional review Board of the different investigation centers (Comité de Protection des Personnes Sud-Est V—Grenoble, France; Comitato Etico dell’Azienda Ospedaliero- Universitaria Policlinico S. Orsola-Malpighi di Bologna, Bologna—Italy; Comitato Etico Ospedale San Raffaele, Milano, Italy; Secreteria del CEIC Area de Salud de Burgos y Soria—Hospital Universitario de Burgos, Soria, Spain; Comité Ético de Investigación Clínica, Hospital Universitario de Alicant, Alicant, Spain; Biomedical Sciences Institutional Review Board Office of Responsible Research Practices, Columbus, USA; Institutional Review Board for Human Research (IRB) Office of Research Integrity (ORI) Medical University of South Carolina, Carleston, USA; Comité D’Etique de la Recherche,Centre Affilié à L’Université de Montreal Hopital Maisonneuve-Rosemont, Montreal, Canada (S2 Appendix). Prior to enrolment in the study, complete detailed information was given to the patient. All patients had to sign an informed consent form, according to local regulations.

### Statistical analysis

General characteristics were tabulated using the mean (minima and maxima) for continuous variables, and number of observations and percentage for categorical variables. Qualitative variables were compared using the Chi^2^ test with a P-value = 0.05.

P-values that compare rates (US vs. OUS) are based on two-sided Fisher's exact test. Multivariate analysis of the effect of clinical variables on PE prevalence was performed by calculating odds ratios (ORs) and their 95% confidence intervals (CI). We created a multivariable logistic regression model with the presence of PE as the outcome. Independent variables included region (USA vs outside USA), age, sex and PTP. We tested for discrimination and fit in the model as well as for the presence of interactions between region and both age and sex.

## Results

A flow chart of the DIET study is presented in S3 Appendix. Patients were included between November 2011 and August 2013 and analyzed in the DIET study primary efficacy analysis. Valid information (i.e., Wells score, D-dimer result, and reference imaging or 3-month follow-up) was available in 1060 cases with low (798 patients, i.e., 75.3%) or moderate (262 patients, i.e., 24.7%) PTP.

### Patients

**Fig 1:**
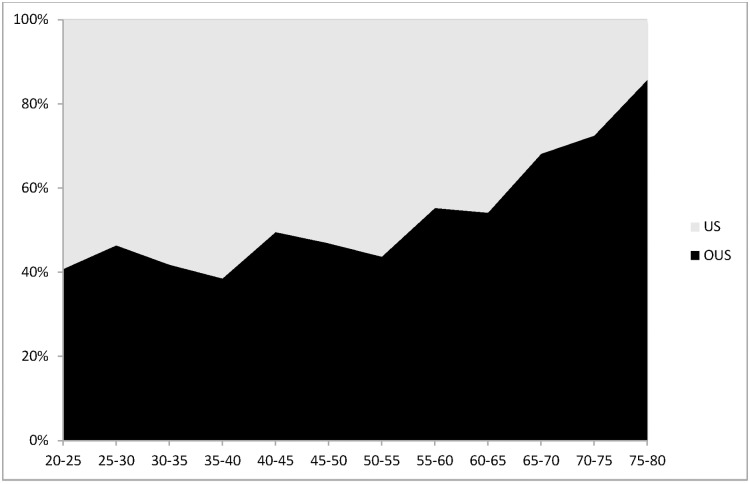
Distribution in age of the DIET population. X axis was age (divided into subgroups of 5 years). Y axis was percentage of population for each category of age. US patients are depicted in grey; OUS patients are depicted in black. P < 0.0001 for comparing the overall differences between US versus OUS.

The patients’ characteristics, divided into US patients (n = 358) and outside US (OUS) patients (n = 702: Canada 80, Europe 622, i.e. 88.6% of the OUS population) are depicted in Table 1. Overall there was a higher proportion of females in the US compared to OUS group. This is true whichever age subgroup is considered (data not shown). The mean age of patients from OUS was nearer 50 years old, while that from the US was nearer 40 years old, and the age distribution differed, with a higher proportion of young patients in the US (Fig 1). The most common symptoms for PE suspicion were “lung-related chest pain (84.5%), dyspnea (50.7%) and hemoptysis (2.4%). The mean Wells score (Table 2) was 1.05 (SD 1.41) and 1.32 (SD 1.66) in the US and OUS respectively. The most frequent Wells clinical score item was tachycardia, both in US and OUS patients. The frequency distribution of PE Wells score items was the same in both groups, with the exception of a higher proportion of “previous VTE” and “PE most likely”in the OUS population (Table 1). Finally, the majority of subjects in both regions belong to low PTP level (80.2% in the US and 72.8% OUS) (P = 0.008). So US has a greater portion of subjects identified as low PTP level as compared to OUS.(Table 2).

**Table 1:** Main characteristics of the patients.

	DIET Study (n = 1060)	US (n = 358) n (%)	OUS (n = 702) n (%)	P (US vs OUS)
Female gender	587 (55.4)	249 (69.6)	338 (48.1)	P < 0.0001
Mean age y (Min—Max)	46.7 (17–79.9)	43.4 (18.8–78)	48.4 (17–79.9)	P < 0.0001
**Wells Score items**				
Immobilization or Surgery In the Previous Four Weeks	33 (3.1%)	17 (4.7%)	16 (2.3%)	P = 0.04
Malignancy (Ongoing Treatment, Treated In the Last 6 Months or Palliative)	0	0	0	
Previous DVT/PE	86 (8.1%)	16 (4.5%)	70 (10.0%)	P < 0.002
Clinical Signs and Symptoms of DVT	37 (3.5%)	10 (2.8%)	27 (3.8%)	P = 0.5
Tachycardia > 100 bpm	226 (21.3%)	89 (24.9%)	137 (19.5%)	P = 0.05
Hemoptysis	26 (2.4%)	7 (2.0%)	19 (2.7%)	P = 0.5
PE most likely	215 (20.3%)	52 (14.5%)	163 (23.2%)	P < 0.001

US, US patients; OUS, outside US patients;

DVT, deep vein thrombosis; PE pulmonary embolism

**Table 2:** Distribution of population and PE prevalence according to Wells Score.

	Population Frequency		PE Prevalence		
Wells Score	US (N = 358) N (%)	OUS (N = 702) N (%)	US	OUS	P (US vs OUS)
0	201 (56.1%)	367 (52.3%)	1 (0.5%)	5 (1.4%)	0.4
1	5 (1.4%)	13 (1.9%)	0	2 (15.4%)	1
1.5	81 (22.6%)	131 (18.7)	2 (2.5%)	15 (11.5%)	0.02
2.5	1 (0.3%)	0	0	0	
3	47 (13.1%)	108 (15.4%)	3 (6.4%)	25 (23.1%)	0.01
4	1 (0.3%)	4 (0.6%)	0	1 (25%)	1
4.5	20 (5.6%)	66 (9.4%)	3 (15%)	20 (30.3%)	0.25
5.5	0	2 (0.3%)	0	1	
6	2 (0.6%)	11 (1.6%)	0	6 (54.5%)	0.46
Low	287 (80.2%)	511 (72.8)	3 (1%)	22 (4.3%)	0.01
Moderate	71 (19.8%)	191 (27.2%)	6 (8.5%)	53 (27.7%)	0.0007

US, US patients; OUS, outside US patients

P value regards PE Prevalence

### PE prevalence

**Fig 2:**
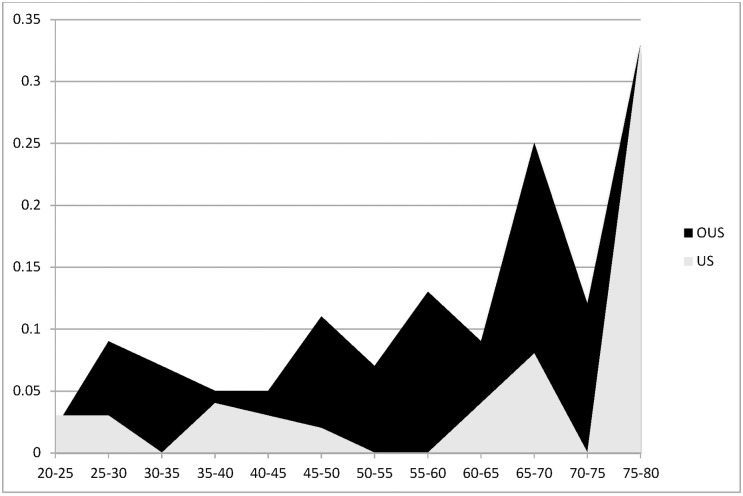
Rate of PE (%) according to age. X axis was age divided into subgroups of 5 years. Y axis was rate of PE (%). US patients are depicted in grey; OUS patients are depicted in black. P < 0.0001 for comparing the overall differences between US versus OUS.

The prevalence of PE in the DIET arm was 7.9% in low and moderate risk patients. Interestingly, PE prevalence was four times higher in OUS (10.7%) than in US patients (2.5%) (P = 0.0001). This was true for each score level and each strata of the PTP, with a PE prevalence of 4.3% in OUS vs 1% in US for low (P = 0.01), and 27.7% for OUS vs 8.5% for US for moderate PTP (P <0.001) respectively (Table 2). In addition, the observed PE rate was systematically higher in OUS across all ages (Fig 2). Since population OUS was mainly from Europe, we tested if excluding Canada-based patients from OUS cohort, and separately including Canada-based patients into the US-based cohort had any impact on the study’s main finding. As shown in Table 3, including or excluding Canadian site from US/OUS population does not affect prevalence results.

**Table 3:** PE Prevalence and negative DD including/excluding Canadian site from US/OUS.

	US	OUS (Europe + Canada)	US + Canada	Europe (w/o Canada)
PE Prevalence (%)	2.5	10.7	3.7	10.9
Negative DD (%)	76	68.8	75.1	68.8

US, US patients; OUS, outside US patients; PE, pulmonary embolism

Interactions between sex and region and between region and age were not significant (data not shown). Therefore, the unadjusted OR for PE in OUS vs US was 4.4 (95%CI 2.2–8.7) (P<0.001), while that after adjustment for age, sex and PTP was 3.4 (95%CI 1.7–7) (p = 0.01).

### D-dimers and imaging procedures

All patients received DD testing. A higher proportion of negative DD was found in the US population (76.0% vs 68.8% for OUS, P < 0.02).

**Fig 3:**
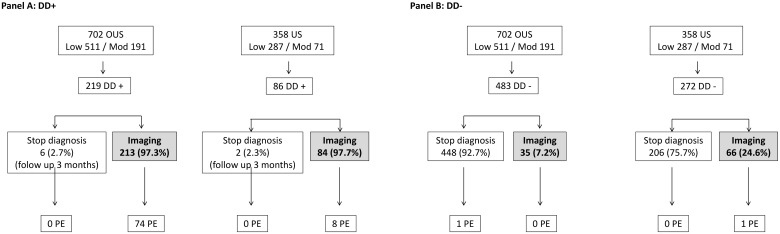
Use of imaging tests in cases of low/moderate PTP combined with positive DD (Panel A) or negative DD (Panel B). US, US patients; OUS, outside US patients; PE, pulmonary embolism; Low, low PTP; Mod, moderate PTP.

For both low and moderate PTP, regardless of DD results, 150 imaging procedures were ordered at US (41.9% of patients) and 249 at OUS (35.5%) sites, with PE diagnosed in 9 and 75 subjects respectively. Therefore, the mean number of CTPA performed for one new PE diagnosis was 3.3 in OUS vs 17 in the US (P < 0.001). Care pattern was strictly the same regarding imaging use in case of positive DD, both in US and OUS. By contrast, stopping investigation in cases of negative DD combined with low/moderate PTP was more frequent in OUS (92.7%) than in the US (75.7%) (p < 0.01) (Fig 3). Moreover, CTPA was used more frequently in the US both in cases of low PTP and negative DD (46/227, i.e. 20.3% in US vs 20/405, i.e. 4.9% in OUS, P<0.001) and moderate PTP combined with negative DD (20/45, i.e. 44.4% in US vs 15/78, i.e. 19.2% in OUS, P<0.01) (Table 4)

**Table 4:** Performance of computed tomographic pulmonary angiography regarding pre test clinical probability combined with negative DDimers.

	OUS	US	
Negative DD with Low PTP	20/405 (4.9%)	46/227 (20.3%)	p < 0.001
Negative DD with moderate PTP	15/78 (19.2%)	20/45 (44.4%)	P < 0.01

US, US patients; OUS, outside US patients; DD, D Dimers; PTP, Pre test clinical probability

## Discussion

This study regarding the use of DD to exclude PE in accordance with new guidelines from the CLSI/FDA showed huge differences in PE prevalence among those evaluated for PE, as well as differences in patients’ characteristics and diagnostic approach between the US and OUS (mainly Europe). This finding is consistent with a previous post hoc analysis of pooled multicenter data, but these were not recorded during the same period [16]. The advantage of the current study is that it prospectively evaluated data in a real-time care setting.

Overall, PE prevalence in this study was much lower than previously described. For example, the PE prevalence in the recent ADJUST PE study published in 2014 was 19% [15]. However the proportion of high PTP patients in the ADJUST PE study was 12.8%, vs 1.6% in the DIET study. This was mainly due to the design of the DIET study. As we were interested in the ability of negative DD to safely exclude PE, we excluded, in accordance with the FDA/CLSI guideline, all patients or situations responsible for possible false positive DD results. Therefore, none of the patients in the DIET was over 80 years old, there was no active cancer (vs 12.9% in the ADJUST PE) and only 3.1% (vs 11.8%) had had surgery in the previous month.

However, although these characteristics might explain the global difference in PE prevalence between these two studies, they do not explain the significant differences between US and OUS PE prevalence in our study. Since the global incidence in the DIET was similar to that previously reported, we concluded that our sample was representative of the PE population in general.

As the incidence of PE classically increases with age, one of the possible explanations for the differences found in this study could be that our OUS patients were older than US. However this did not seem to be the underlying cause, because a higher rate of PE was observed in OUS regardless of age (Fig 3). Another characteristic of the OUS population was that the proportion of patients classified as “moderate PTP” was higher (27.2%) (Table 2). This might be explained by characteristics such as a higher proportion of “previous VTE” in OUS (giving a Wells score of 1.5), and also by the fact that physicians in Europe considered PE as the “most likely diagnosis” more often than those in the US (which scored 3) (Table 1). Interestingly, the item “PE most likely” was recently described as one of three Wells score items that significantly added incremental value to the DD test [19]. These characteristics of patients with a higher proportion of moderate score might explain the higher prevalence in OUS. However, it cannot be the only explanation because the relative prevalence (x 4) in the OUS vs US was similar in both low and moderate PTP, and also the difference in prevalence persisted even when controlling for population characteristics including age, gender. This suggests that besides patients’ characteristics, differences in care patterns might also explain the differences seen. The above indicates that physicians in the US and OUS behave similarly with respect to the PTP parameters. However US physicians have a “lower threshold” for suspecting PE than non-US physicians. This probably means that US EDs were evaluating for PE even when they thought it unlikely, which caused them to suspect more patients of having PE. Therefore the “Total N” in the equation (n with PE/Total N) would be much larger in a US emergency department, leading to a smaller prevalence.

The diagnostic approach also differed between US and OUS sites. Although DD had similar test characteristics (sensitivity, specificity and likelihood ratios) in the two regions, US physicians performed CTPA 3 to 5 times more often than in Europe in cases of low/moderate PTP, consistent with a previous report [16] (Fig 3). This might reflect in part the perception and adherence of physicians to PE diagnosis guidelines. In cases of moderate PTP, we showed through the DIET study that most clinical centers in the US do not usually prescribe the D-dimer test, but favor direct imaging. Regional differences between PE diagnosis guidelines might explain the different practices. In Europe, the European Society of Cardiology [7] proposed stopping further investigations in cases of low or moderate PTP combined with highly sensitive negative DD, with a high 1A grade of recommendation. Similar recommendations were recently made by the American College of Physicians [20]. In contrast, the American College of Emergency Physicians (ACEP) [10] recommended this approach only in cases of low PTP (grade A), but with extreme precaution in cases of moderate PTP (grade C). The ACEP argued that, despite consensus guidelines that recommend using DD on patients with intermediate PTP for PE, strong evidence supporting this approach is lacking. Therefore US practitioners favor suspecting PE and initiate a diagnostic process at a very low threshold of symptoms. This translates into an increase in the number of imaging tests used, even in cases of low/moderate PTP with negative DD, for which US practitioners used CTPA four times more frequently than in Europe. This approach remains subject to debate however, since in cases of low/moderate PTP with negative DD, the rate of non-diagnosed observed PE was only 0.2%, much lower than that observed when applying pulmonary embolism rule-out criteria (PERC) to low PTP to exclude PE without further testing [21], or when PE was excluded based on negative CTPA [22]. This apparent over investigation, especially involving the use of CTPA, might lead to a low prevalence, but also radiation overexposure and possibly over diagnosis.

## Conclusion

In this study, the perception and adherence of physicians to PE diagnosis guidelines explained the huge difference observed in PE prevalence among PE-suspected patients admitted to emergency units. This was accompanied by the overuse of CTPA by US practitioners, especially in cases of moderate clinical probability, in order to exclude PE.

## Supporting Information

S1 Appendix Characteristics of centers participating to the Diet Study. (TIF)

S2 Appendix List of ECs or IRBs For the study with the title: “STA^®^—Liatest^®^ D-Di—Exclusion of Venous Thromboembolism (VTE)”. Brief title DiET.
(TIF)

S3 Appendix Flow chart of the DIET PE study. PE, pulmonary embolism; PTP, pretest probability (Wells score).
(TIF)
