# Supplementary figures and images for: D-Dimer Use and Pulmonary Embolism Diagnosis in Emergency Units: Why Is There Such a Difference in Pulmonary Embolism Prevalence between the United States of America and Countries Outside USA?
Source: PLoS One. 2017 Jan 13;12(1):e0169268. doi: 10.1371/journal.pone.0169268 (PMC5234786; doi:10.1371/journal.pone.0169268)

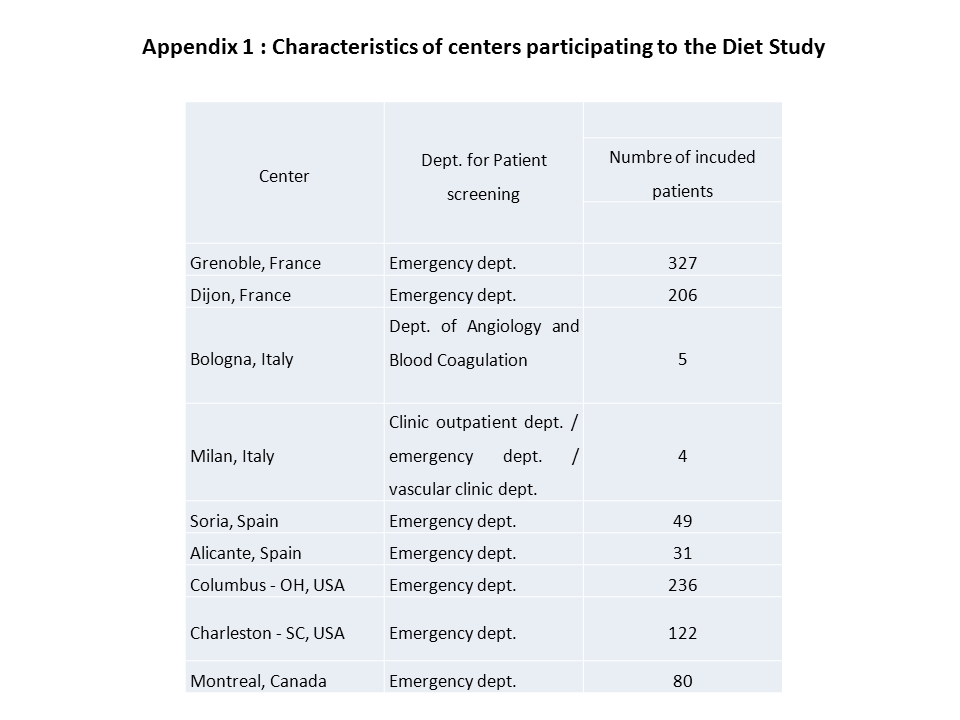

Supplement: S1 Appendix — (TIF) [file pone.0169268.s001.tif]

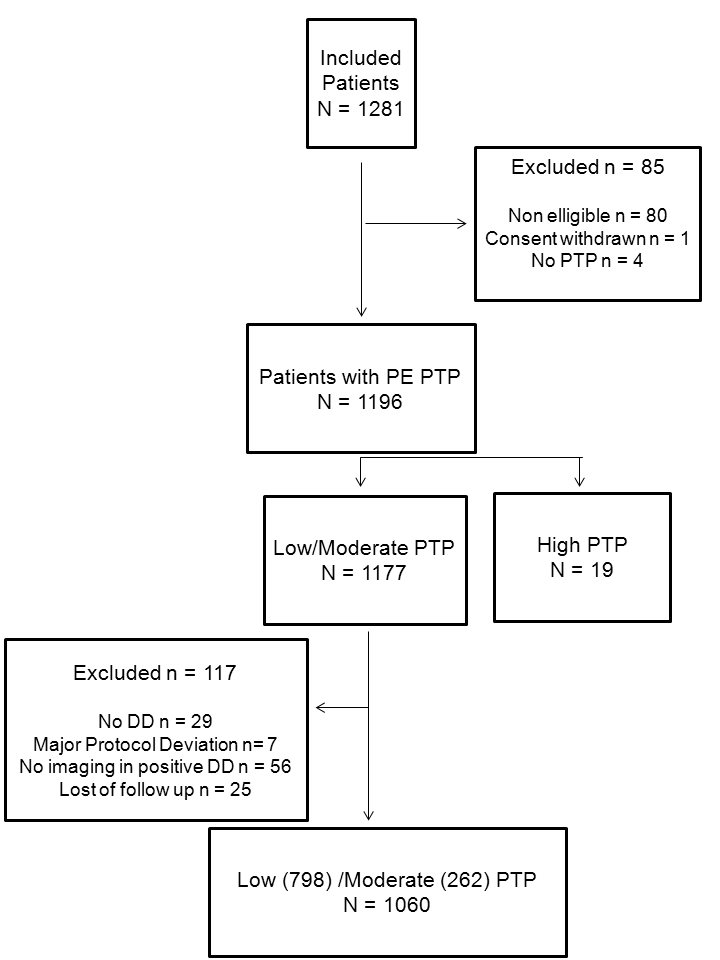

Supplement: S2 Appendix — Brief title DiET. (TIF) [file pone.0169268.s002.tif]

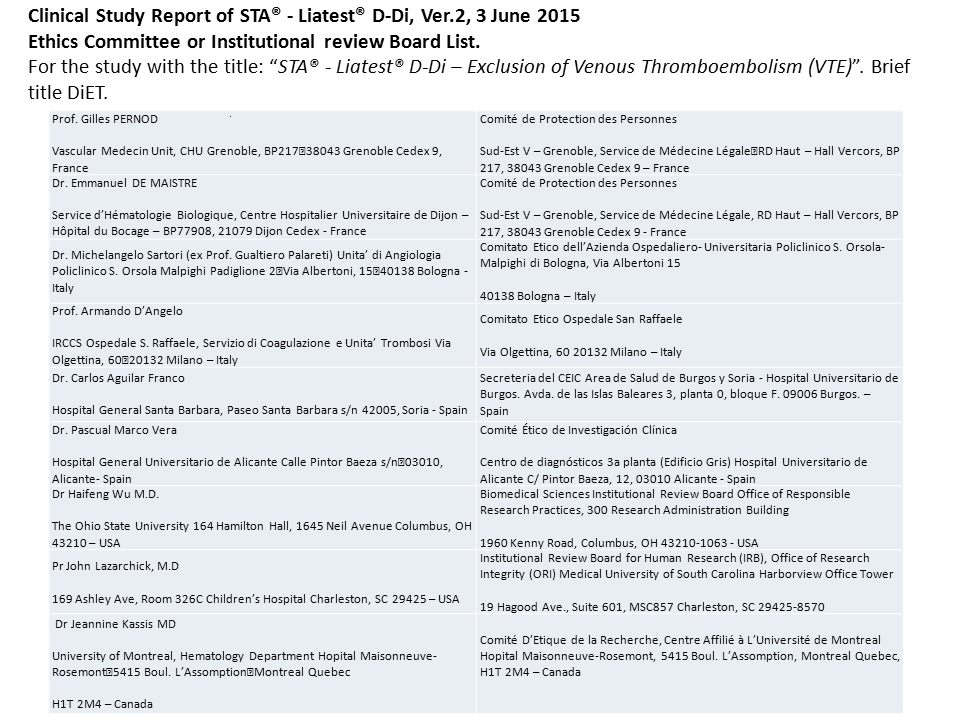

Supplement: S3 Appendix — PE, pulmonary embolism; PTP, pretest probability (Wells score). (TIF) [file pone.0169268.s003.tif]
